# Omics and Bioinformatics Approaches to Identify Novel Antigens for Vaccine Investigation and Development

**DOI:** 10.3390/vaccines8040653

**Published:** 2020-11-03

**Authors:** Jorge H. Leitão, Manuel J. Rodríguez-Ortega

**Affiliations:** 1 iBB—Institute for Bioengineering and Biosciences, and Bioengineering Department, Instituto Superior Técnico, Universidade de Lisboa, 1049-001 Lisbon, Portugal; 2 Department of Biochemistry and Molecular Biology, University of Córdoba, Campus de Excelencia Internacional CeiA3, 14071 Córdoba, Spain; mjrodriguez@uco.es

**Keywords:** omics, genomics, proteomics, antigen identification, bioinformatics tools, subunit vaccine, high-throughput techniques

## Abstract

Despite the outstanding technological advances achieved in the last few decades, infectious diseases remain a major societal challenge. From the variolation carried out in ancient China during the 15th century to the more advanced RNA and DNA vaccines presently available, vaccines have been proven as highly effective therapeutic tools to combat various infectious diseases. Vaccine research and development is now empowered with recent advances in Omics sciences and the developments of powerful bioinformatics tools. This Special Issue has gathered a total of nine original papers, including seven research papers and two reviews, illustrating the use of Omics data and bioinformatics in the research, design and development of vaccines against pathogens and cancer. The integration of knowledge from Omics and Bioinformatics will certainly boost vaccine research and development, leading to novel therapeutic tools against new and old pathogens and cancer in the near future.

Infectious diseases remain a major societal challenge, enhanced by global threats, including the emergence of multidrug resistant pathogens due the misuse and abuse of antimicrobials, climate change and the associated emergence and re-emergence of new and old pathogens, and the easy and fast spread of infectious diseases at a global scale. From the variolation carried out in the 15th century in ancient China to the more advanced RNA and DNA vaccines available in recent years, vaccines have been proven as highly efficient in conferring protection to various infectious diseases. In addition, recent developments in the field of cancer immunotherapy are leading to advances in vaccines against specific cancers. The fast-developing field of Omics sciences is delivering a deluge of nucleotide and protein sequence data, which is impossible to exploit without powerful bioinformatics tools and databases. This Special Issue was launched with the aim of gathering contributions combining the uses of post-genomics and bioinformatics in vaccine design and development. A total of nine original papers were published, including seven research papers and two reviews.

As this editorial is being written, on the 19 October 2020, more than 40 million infections and 1.2 million fatalities have been registered worldwide according to the COVID-19 Dashboard from the Center for Systems Science and Engineering (CSSE) at Johns Hopkins University [1]. An unprecedented worldwide race for protective vaccines against the SARS-CoV-2 virus is ongoing, and at least a hundred vaccine candidates are under development, with some already in the phase of human trials. In their paper, Mukherjee et al. [2] point out the critical requirement of using epitopes capable of stimulating both B-cell and T-cell protective responses to design an effective vaccine against the SARS-CoV-2 virus. The authors extensively used bioinformatics tools to predict the occurrence, in the proteome of the virus, of potential immunogenic epitopes. They considered the identified epitopes as immunodominant when three criteria were met: when they were predicted as both B- and T-cell potential epitopes, when they were predicted as protective, and when they perfectly matched experimentally validated SARS-CoV-2 epitopes [2]. The authors also performed structural and molecular docking analyses to gain a further understanding of the binding interactions established by the predicted immunodominant epitopes with the major histocompatibility complexes of the human host [2]. The set of SARS-CoV-2 immunodominant epitopes predicted to be capable of inducing both a humoral and cellular-mediated immunity constitutes an important contribution towards the design and development of vaccines to prevent the ongoing COVID19 pandemic and future outbreaks caused by the virus.

The *Burkholderia cepacia* complex (Bcc) comprises at least 24 distinct but closely related bacterial species capable of causing severe and life-threatening infections [3]. These bacteria have attracted particular attention since the early 1980s due to their ability to cause debilitating and often fatal respiratory infections to patients suffering from cystic fibrosis (CF). Despite the remarkable advances in CF therapy, Bcc respiratory infections remain difficult, and often impossible, to eradicate using clinically available antibiotics, and no protective vaccines are available [4]. Aiming at the identification of surface-exposed proteins of potential use as components for the development of vaccines protective against Bcc infections, Sousa et al. used a strategy combining bioinformatics to identify putative immunogenic surface-exposed proteins with the experimental identification of surface proteins by “shaving” live bacteria with trypsin, followed by liquid chromatography and mass spectrometry [5]. The “shaving” methodology was developed by Rodríguez-Ortega et al. to identify surface-exposed streptococcal proteins as potential candidates for vaccine development [6]. Sousa et al. validated the results by demonstrating the immunoreactivity of three surface-exposed proteins with serum samples from CF patients with a clinical record of infection by Bcc [5]. Since Bcc bacteria share with other pathogens of the *Burkholderia* genus, such as *B. pseudomalllei* and *B. mallei*, a large number of conserved proteins, the authors foresee that the exploitation of Bcc immunogenic proteins might lead to the development of a broad-spectrum vaccine protective against infections by pathogens of the *Burkholderia* genus [5].

A “shaving” approach was also used by Galán-Relaño et al. to identify the pan-surfome (i.e., the complete set of surface proteins) of *Trueperella pyogenes* [7]. The bacterium is an opportunistic pathogen in both wild and domestic animals, causing suppurative infections that may be severe, with mortality rates that increase due to misdiagnosis or inadequate treatment [8]. The pathogen can cause significant economic losses in swine production, and no commercial vaccines are available [7]. In their work, the authors used a collection of 15 clinical isolates and performed the digestion of live isolates with trypsin, and identified a total of 140 surface proteins [7]. After ranking the identified proteins based on their surface-exposed location, conservation and distribution among *T. pyogenes* isolates, the authors identified in more than 70% of the isolates under study a set of two cell wall proteins, three lipoproteins, four secreted proteins and seven membrane proteins. The identified proteins were considered as potential candidates for effective vaccines or for diagnostic tools of *T. pyogenes*.

Prados de la Torre et al. also used the “shaving” approach to identify surface proteins from six *Streptococcus suis* human clinical isolates of the major serotype 2 [9]. The microorganism is a Gram-positive commensal of the upper respiratory tract of pigs, but can cause severe infections in animals when in the lower respiratory tract, as well as other invasive and often lethal infections. The bacterium is also an emergent zoonotic pathogen, mainly affecting humans in close contact with infected animals or consumers of raw or under cooked animal products, and no protective vaccine is available [10]. The authors identified a total of 131 *S. suis* predicted surface proteins, and their relative abundances in the studied human isolates were assessed. The potential immunogenicity of selected transmembrane proteins was also assessed using bioinformatics tools, thus contributing to the identification of novel candidates to develop a vaccine against this zoonotic pathogen [10].

Fungal infections are emerging worldwide, but the number of therapeutic options is restricted and there is a steadily increasing number of fungal clinical isolates resistant to multiple drugs [11]. Although the fungal pathogens best known belong to the *Candida* genus, *Lomentospora prolificans* is an emerging fungal pathogen with the remarkable ability to enter the blood stream and cause disseminated infections [12]. The situation is of particular concern in severe immunosuppressed patients, particularly in those suffering hematological malignancies [12]. No effective therapeutic treatments are available to treat *L. prolificans* infections. Buldain et al. carried out murine disseminated infections with *L. prolificans* and other fungal species (*Scedosporium aurantiacum, S. boydii and Aspergillus fumigatus*). *L. prolificans* was the most virulent fungal species under study, with high fungal loads in several organs of the animals, including the brain [12]. The authors used the sera from infected animals to identify *L. prolificans’* most immunoreactive proteins. The results presented indicate a high cross-reactivity between *Scedosporium* sp. and *L. prolificans*. Several antigens from *L. prolificans* were identified and considered as potential candidate drug targets, and were exploitable for the development of diagnostic methods and protective vaccines [12].

The worldwide increase in the resistance of pathogens to available antibiotics poses tremendous challenges. This is also the case of the sexually-transmitted *Neisseria gonorrhoeae*, for which the development of protective vaccines is hampered, among other factors, by suitable antigen scarcity and adequate animal models of infection. Zhu et al. designed a new strategy for antigen identification based on reverse vaccinology and bioinformatics, termed the candidate antigen selection strategy (CASS) [13]. Using previously identified pools of proteins expressed by *N. gonorrhoeae* during human mucosal infections, the authors identified 36 membrane-associated proteins that were conserved in *N. gonorrhoeae* and predicted as immunogenic [13]. Out of the 36 candidates, 6 were used to immunize mice, and were shown to be capable of inducing cross-reactive antibodies and serum bactericidal activity. The results suggest the CASS strategy as a useful tool in vaccine candidate identification [13].

Dar et al. reported an in silico strategy to identify the antigenic proteins of *Klebsiella pneunoniae*, an opportunistic pathogen responsible for nosocomial infections [14]. The authors performed data mining of the pangenome of *K. pneumoniae*, resulting from 222 complete genome sequences available at NCBI, to obtain the core proteome. After an initial selection of 35 proteins based on their predicted localization as outer membrane or extracellular proteins, 4 were selected for immunoinformatics studies using the VacSol bioinformatics tool [14]. The four proteins were selected based on their predicted association with virulence, lack of homology with human proteins, and reduced number of transmembrane helices. Using a reverse vaccinology strategy, several bioinformatics tools were used to prioritize the B-cell derived T-cell epitopes. The authors then designed a multi-epitope vaccine composed of a total of 230 amino acids, containing the epitopes linked by GPGPG flexible links and a EAAAK linker to the Cholera Toxin Subunit B coadjuvant. Further in silico studies were conducted by the authors, including the physicochemical evaluation of the multiepitope vaccine, the modeling of its 3D structure and molecular dynamics simulations, and molecular docking studies with Toll-like receptors, as well as reverse translation and codon optimization [14]. The in silico-engineered multiepitope vaccine has the potential to trigger both successful humoral and cellular immunity responses to *K. pneumoniae*, but experimental validation is required to demonstrate its effectiveness as a protective vaccine against the pathogen [14].

In their review, Lucchesi et al. presented an overview of computational tools available for the analysis of data from flow cytometry in vaccination studies [15]. Flow cytometry, and in particular multiparametric flow cytometry, are adequate for the in-depth analysis of immune responses to vaccination. The authors reviewed the usefulness and applicability of several bioinformatics tools, from data pre-processing to automated data analysis and the interpretation of results [15]. The authors also reported the increasing number of publications using the automated analysis of multidimensional cytometry data, based on searches on the Web of Science, and identified a rising trend starting in 2008, with a stronger increase in the period 2014–2019 [15]. The automated analysis of data from cytometry has already proven to offer reliable and reproducible results. The integration of these data, from a systems biology perspective, with data from Omics approaches (genomics, transcriptomics, proteomics, metabolomics) and also with clinical data, will certainly lead to a better understanding of the responses of the immune system to antigenic challenge, as is the case of vaccination [15].

While most of the contributions to this Special Issue have focused on vaccines against pathogens, Lokhov et al. reviewed the research work and developments achieved with SANTAVAC^TM^, an antigen composition under development using a strategy combining proteomics and cell culture technologies, envisaging the production of vaccines against several solid tumors [16]. The SANTAVAC^TM^ vaccine was designed to target tumor vessels, and is expected to outperform the approved antiangiogenic drugs available on the market [16]. The research and development of vaccines against cancer is a relatively recent, but highly active research field, expected to contribute to the cure of various types of cancers in the near future. The review describes the progress achieved so far with SANTAVAC^TM^, and summarizes the promising results obtained in non-clinical studies [16]. The research and development phase of SANTAVAC has been concluded, and the vaccine is in the preclinical stages [16].

The collection of papers published in this Special Issue highlight the intense activity of the research into and development of vaccines towards human pathogens, including viruses, bacteria and fungi, as well as the exploitation of knowledge from cancer immunobiology, to develop cancer vaccines. The remarkable advances achieved in this exciting research field would not have been possible without the accompanying developments of powerful bioinformatics tools and the available Omics data. The integration of knowledge from these scientific fields is expected to boost vaccines research and development, leading to novel therapeutic tools against pathogens and cancer in the years to come.
